# Osteochondritis dissecans (OCD) in Horses – Molecular Background of its Pathogenesis and Perspectives for Progenitor Stem Cell Therapy

**DOI:** 10.1007/s12015-019-09875-6

**Published:** 2019-02-23

**Authors:** Lynda Bourebaba, Michael Röcken, Krzysztof Marycz

**Affiliations:** 1 Department of Experimental Biology, Faculty of Biology and Animal Science, Wrocław University of Environmental and Life Sciences, Norwida 27B, 50-375 Wrocław, Poland; 2 Faculty of Veterinary Medicine, Equine Clinic - Equine Surgery, Justus-Liebig-University, 35392 Gießen, Germany

**Keywords:** Osteochondrosis, Dissecans, Cartilage, Regenerative medicine, Stem cells, Molecular events, Chondrocyte

## Abstract

Osteochondrosis (osteochondrosis dissecans; OCD) is a disease syndrome of growing cartilage related to different clinical entities such as epiphysitis, subchondral cysts and angular carpal deformities, which occurs in growing animals of all species, including horses. Nowadays, these disorders are affecting increasing numbers of young horses worldwide. As a complex multifactorial disease, OCD is initiated when failure in cartilage canals because of existing ischemia, chondrocyte biogenesis impairment as well as biochemical and genetic disruptions occur. Recently, particular attention have been accorded to the definition of possible relations between OCD and some metabolic disorders; in this way, implication of mitochondrial dysfunctions, endoplasmic reticulum disruptions, oxidative stress or endocrinological affections are among the most considered axes for future researches. As one of the most frequent cause of impaired orthopaedic potential, which may result in a sharp decrease in athletic performances of the affected animals, and lead to the occurrence of complications such as joint fragility and laminitis, OCD remains as one of the primary causes of considerable economic losses in all sections of the equine industry. It would therefore be important to provide more information on the exact pathophysiological mechanism(s) underlying early OC(D) lesions, in order to implement innovative strategies involving the use of progenitor stem cells, which are considered nowadays as a promising approach to regenerative medicine, with the potential to treat numerous orthopaedic disorders, including osteo-degenerative diseases, for prevention and reduction of incidence of the disease, not only in horses, but also in human medicine, as the equine model is already widely accepted by the scientific community and approved by the FDA, for the research and application of cellular therapies in the treatment of human conditions.

## Introduction: Overview

Developmental diseases of the equine skeleton, which include any disorder (inherited or acquired) that can interfere with normal development, modelling or remodelling of bone, involve a wide range of different conditions that may manifest as a partial or complete failure during early stages of bones or limbs development, or as chondrodysplasias and osteodysplasias that will affect the whole skeleton. Of the many conditions recognized to occur in the horse, osteochondrosis is considered to be manly associated with the complex of “developmental orthopaedic diseases” [1].

Osteochondrosis (OC) is a multifocal pathology, which takes place in both articular-epiphyseal cartilage complex (immature joint cartilage covering the ends of growing long bones) and growth plate in a variety of mammalian species. The disorder is characterized by failure of endochondral ossification, and is considered as one of the most common primary causes of degenerative joint disease in domestic animals [2, 3]. One of the most recurrent manifestations of OC is the *osteochondrosis dissecans* (OCD), which is considered to be involved in failure of cellular differentiation in growing cartilage, leading to its dramatic thickening or retention, emergence of fissures and eventual focal loss of cartilage flaps into the joint cavity. The detached fragments can be responsible of severe joint inflammation, which can lead to subsequent development of secondary osteoarthritis (OA) [4].

Initially, the term ‘osteochondritis dissecans’ was first introduced by the German surgeon Franz Konig (1832–1910), when he conducted a study on loose bodies in joints; he suggested three possible underlying causes for the disease. The first two were of traumatic origin including sever fragmentation or subchondral bone necrosis, which indirectly leads to fragments loss. The third category lesions were reported to appear without any significant trauma, and seemed to be related to some other factors [5]. OCD was then reported again in 1947 in horses as abrasion of the lateral trochlear ridge with lateral patellar luxation. Thereafter, similar lesions were observed and reported as being osteochondral fractures- and osteochondritis dissecans. In 1968, intracapsular bony fragments of the distal tibia were diagnosed in several horses, and described later as OCD of the tibiotarsal joint and surgical removal of fragments was reported to be a beneficial treatment [6]. Later, researches established that this lesion was a focal disturbance of the endochondral ossification process. Nowadays, it is this latter definition that is widely used in veterinary literature [5].

Because this process is typically only active in horses during the first year after birth, osteochondrotic lesions, by definition, will only develop during this period. The tarsocrural joint is one of the joints most commonly affected by osteochondrosis or OCD in warmblood horses, and lesions at this location often do not clinically manifest until later in life, when training commences and young horses are first subjected to athletic challenges [7]. It was reported that young Arab and Quarter horses are frequently subjected to development of cystic lesions of the weight bearing medial femoral condyle; however, the condition is much rarer in ponies since only few cases were recorded. An estimated 20–25% of new-born foals will develop some form of OCD. Statistics showed that in northwestern Europe alone, OCD affects 20,000 to 25,000 foals every year. OCD is therefore one of the most important of the so-called ‘developmental orthopaedic diseases’. More precisely, the youngest foal found with dissecting lesions was a 3-day-old Standardbred colt. Then, 3 months and up to 6 months old foals were also diagnosed for OCD. Furthermore, it was noted that the degree of severity of the dissecting lesions conditioned the age at which, the first clinical signs of the disease start to appear, since it has been shown that the most moderate lesions were in most cases undetectable. It has also been observed that the development of lesions in Thoroughbred and Half-bred horses was faster and more obvious than in trotting horses [8, 9].

It is now well established that Osteochondritis dissecans (OCD) is a major problem in the equine industry. In fact, OCD or what can be described as failure of normal cartilage maturation, is the predominant cause of impaired orthopaedic potential, whose symptoms may be of minimal magnitude or manifest as severe joint effusion or clinically noticeable lameness [1]. In competition horses, lameness is the direct consequence of osteochondrosis lesions that dramatically reduce the athletic performance of affected horses. This strongly hampers the racehorses and generates significant veterinary costs resulting in serious economic losses in all sections of the equine industry [10]. Moreover, particular attention to OCD detection is usually given when selecting horses that will be dedicated for breeding future generations [11]. Since bone and joint disorders are the main limiting factors affecting the horse industry through loss of breeding potential and reduced market value, it would therefore be important to first add further data on the pathology and the underlying risk factors, as well as to develop possible new strategies to enhance the management of OCD in horses [12]. Thus, as a part of the development of innovative therapies for the management and treatment of various orthopaedic conditions, the horse seems to represent an experimental model of choice, mainly because of the high similarity of its cartilage to that of human beings, from a structure, thickness and cellularity point of view, and offers great opportunities for advancing research in terms of understanding and developing new preventive and curative therapies [13].

## Endochondral Ossification in Health

**Fig. 1:**
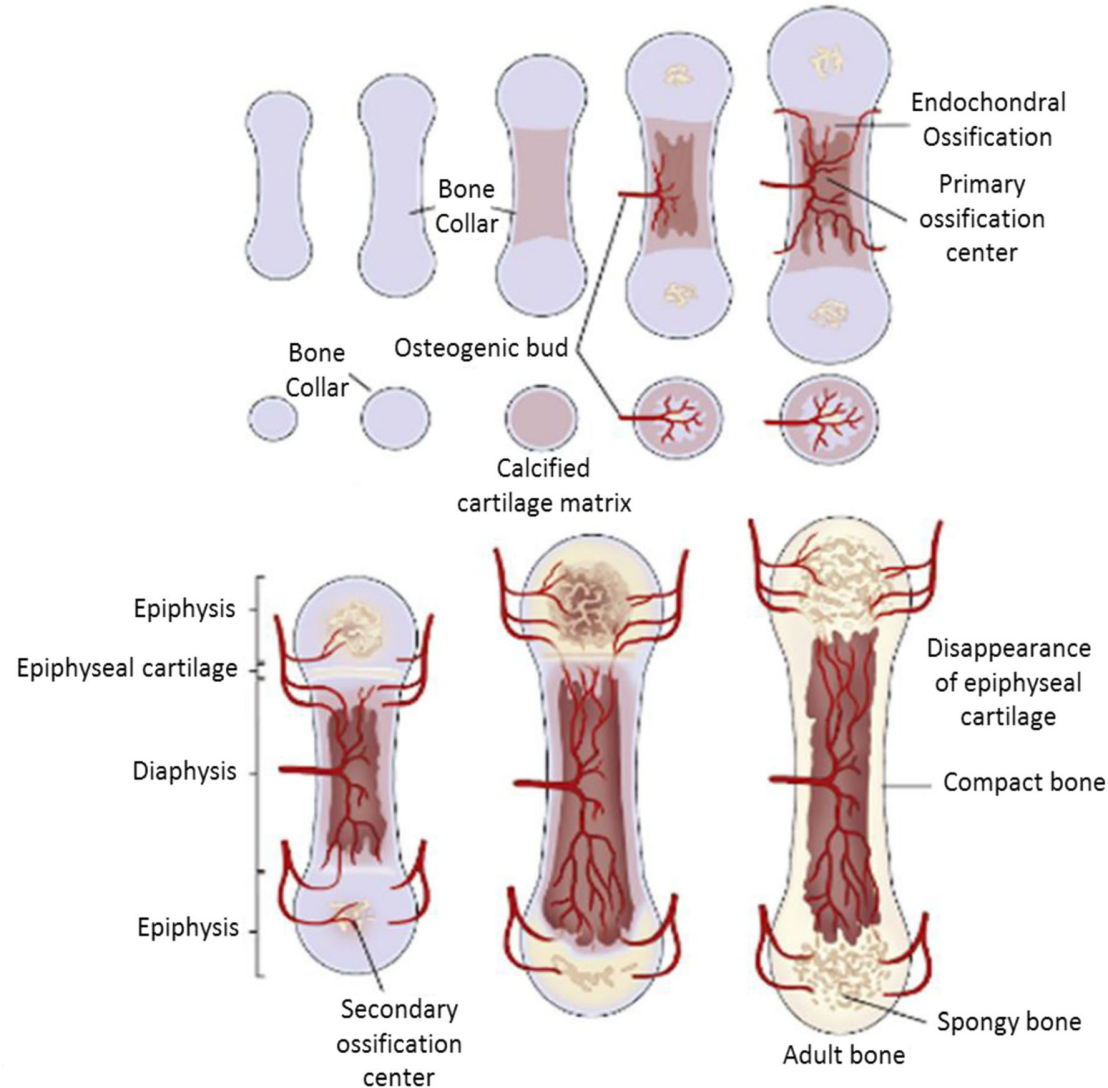
Ossification process of the long bone in mammals [19]

During the development of the equine skeleton, cartilage growth is a decisive process with regard to the many disorders that can be generated when certain anomalies occur [14]. During the first few months after birth, foals grow quite rapidly, however the rate of growth slows down when they advance in age. It has also been shown that cartilaginous growth plates of the different bone parts close at different times during the skeleton development [15]. Articular cartilage covers the ends of long bones, preventing their surface’s frictions and allowing normal movement of joints. In the postnatal period, the process of growing foals engenders dramatic changes in articular cartilage and the underlying epiphyseal cartilage. The epiphyseal cartilage is transformed into bone, whereas articular cartilage remains cartilage throughout the horse’s life (Fig. 1). This phenomenon manly includes endochondral ossification and cartilage canal formation and regression [16]. The condensation and differentiation of mesenchymal cells into chondrocytes is responsible for the formation of a so-called cartilaginous template and the subsequent development of the diarthrodial joints bones [17]. Endochondral ossification takes place when cartilaginous tissue starts to be gradually replaced by bone tissue. The initiation of this process is ensured by a primary ossification center located in the middle of the cartilaginous bone. Later, secondary ossification centers will develop in the bony ends, triggering the modelling of the subchondral plate and calcified cartilage. Finally, the ossification at the distal parts of the bone stops just before all the cartilage has been calcified, thus allowing the development of the articular cartilage from the remaining cartilaginous tissue [18]. Equine cartilaginous canals that nourish chondrocytes, which are not in direct contact with the nutrient-rich synovial fluid consist of three main sections. The first section so-called proximal, receives its arterial source from the perichondrium throughout growth. The middle part and the distal terminuses initially bifurcate from the arterial perichondrium. Nevertheless, with the ossification extent, these two parts will consecutively anastomose and begin to receive blood from the vessels of the subchondral bone. This constitutive joint-dependant regression represents a normal physiological aspect of the cartilage canals in horses and pigs [17].

If articular cartilage begins to degenerate and moreover to detach from the bone surface, an osteochondrosis dissecans (OCD) condition settles, and an associated lameness eventually develop. A number of interfering and triggering factors have been associated with the pathogenic process of dyschondroplasia; however, very little information regarding the first steps leading to the onset of primary lesions characteristic of the condition are currently available, due to late onset of early clinical signs in affected animals [20].

## Aetiology

Osteochondrosis (OC) is considered as the initial process of a developmental disease, which ultimately results in osteochondral fragmentation and consequent development of OCD. When early lesions of OC appear, they will tend to either heal spontaneously, or to evolve to a more advanced stage, resulting in the appearance of dissecting lesions characteristic of OCD [16].

Involvement of diet, growth rate, certain hereditary factors and trauma in orthopaedic disorders manifestations makes OCD a multifactorial disease. Usually, young horses with rapid growth rates, in addition to genetic and environmental influences, are frequently predisposed to develop OCD pathology [19]. Indeed, it has been shown in some comparative studies conducted on adult subjects who showed a rapid growth rate during their development and characterized by a large musculature and low fat mass, that they were much more exposed to development of OCD as opposed to slower-growing horses, which were much less likely to develop the disease. Moreover, these differences in susceptibility to OCD were also reflected on the offspring of certain males in the same breed. In addition, the increased consumption of high energy feed has long been recognized as a major risk factor in the acquisition of OCD. Thus, restricting caloric intake of foals at an early age has been reported to be beneficial for slowing down the growth rate and consequently decreasing the frequency of osteochondrosis development [8]. The theory that trauma is one of the main etiological causes of OCD development, was one of the first that have been proposed. This has been largely supported by the characterization of chronic gross and histological lesions in the formed osseocartilaginous flap, and the location of these lesions mainly in locations of increased biomechanical stress. Furthermore, it has been speculated that epiphyseal cartilage is intrinsically weaker and more susceptible to trauma than articular cartilage [3]. Nevertheless, traumatic factor does not explain all aspects related to OC, and the vascular aetiology seems to provide more details on the subject. In fact, it is well known that epiphyseal tissue of adult horses becomes avascular at maturity, this may explain why osteochondrosis only occurs in young foals during their period of skeletal growth [2]. In horses, the development of certain abnormalities of the cartilaginous canal blood vessels causing ischemia and local chondronecrosis, were correlated with appearance of early osteochondrosis lesions of the epiphyseal-articular cartilaginous complex, making ischemia a probabilistic risk factor for the development of OC(D) [21].

Genetic predisposition is one of the predominant risk factors in the development of any type of OC including OCD. Thus several cases of OC have been reported to be genetically transmitted in Human; further study of the pathology in twins revealed the presence of identical lumbar spine lesions [22]. In horses, it was observed that non-breeds were significantly less affected by OCD compared to domestic breeds (about 2.5% in the tarsocrural joint and even 0% in the patellofemoral joint). Moreover, almost no cases of OCD were diagnosed in ponies, unlike the high incidence found in many other horses’ breeds [5]. More obviously, the breed considered to be the most predisposed to OCD is the Thoroughbred, followed by Standardbred breeds and Warmblood horses. More recently, clinical signs of OCD have also been found in the coldblood breed [23], which until now were considered to be non-susceptible to the disease. Thus, the genetic origin of OCD has been estimated over a very wide range of heritability coefficient values ranging from 0.07 to 0.65 [24]. Recent investigations have estimated rates of heritability for OC(D) in the order of 0.16 to 0.17 for fetlock, varying between 0.35 and 0.46 for the OCD affecting the hock and ranging from 0.21–0.23 for stifle in Hanoverian warmblood population. Similar results for Standardbred Norwegian trotters were also generated for OCD reaching the distal mid-tibial crest and the trochlear side crest of the talus [25, 26]. Many reports have already been issued and it seems to be proved that the genetic background of that disorder is complex and controlled by a large number of genes. Indeed, alteration of several genes’ expression involved in the process of normal cartilage development has been detected. Horses that have been fed with energy-dense diet for several weeks and have pre-existing OCD lesions, have shown significant overexpression of the encoding genes for extracellular proteins, proteins with an extracellular matrix turnover and cells signalling factors (cathepsin K, LRP4, integrin αV, osteopontin, lumican, ATP6V0D2, thymosin b4 and IBS) leading to a fundamental defect that induces cartilage retention in the subchondral bone. Moreover, an abnormality in the expression of genes encoding for MMP13 (metallopeptidase 13 matrix) and the Runx2 factor (transcription factor linked to runt 2) in the damaged cartilage was also detected [27]. Study of cartilage samples withdrawn from Standarbred foals’ hock joints with OCD lesions, which were descendants from two parents also suffering from the disease revealed an alteration of the TLK2 gene (ruffled kinase 2) and an unknown gene (CD465746.1), in comparison to the cartilage of healthy foals [28]. A comparison of the gene expression in young Belgian Warmblood horses with and without OC, showed that there was a significant alteration in the expression of certain genes involved in the differentiation processes of cartilage and endochondral ossification in the case of OC horses. The same study, identified defects in expression of the PDGF-A (platelet derived growth factor), PTH-rP, Wnt / b-catenin genes and Ihh (Indian hedgehog), suggesting that metabolic disorders such as insulin resistance could take part in the pathogenesis of OC [29]. The emergence of microRNAs appeared to be potentially new biomarkers for the study of OC/OCD [30]. Many microRNAs have been identified in recent years as playing a crucial role in the regulation of various processes during osteochondral ossification. Their overexpression or repression generally result in pathological modification of the transcription patterns of key genes of the cartilage components. For example, down-expression of mir-140 leads to progressive loss of proteoglycan as well as cartilage fibrillation, which are characteristic of early osteoarthritis [31]. Moreover, the absence of this microRNA, induces the activation of the key enzyme Adamts-5, which is responsible for cartilage degradation during OC pathogenesis. Other results have also shown that overexpression of miR-145 results in a suppression of Sox9 expression, that is a key factor in the chondrogenic differentiation of mesenchymal stem cells. This results in a collapse of the expression of the genes encoding for Col2A1, ACAN, Comp, Col9A2 and Col11A1; and upregulation of genes associated with hypertrophy, namely Runx2 and MMP-13 [32]. More recently, analysis of miRNAs in synovial fluid samples obtained from French trotters has been performed; the results of the gene expression monitoring could reveal no less than ten physiopathological pathways that may be involved in the development of OC, mainly with alterations in the PI3K / Akt, TGF-β, insulin, MAPK and calcium signaling pathways, but also endocytosis and focal adhesion [33].

## Cartilage Derived Stem/Progenitor Cells (CSPCs)

Cartilage is a tissue characterized with a low self-regeneration potential when it is damaged, since it is avascular, aneural, and blood circulating there is poorly concentrated in different growth factors that are necessary for cellular multiplication and regeneration. [34]. Indeed, the cartilaginous tissue is characterized by a simplified structuring, comprising only one cell type (chondrocytes) and an abundant ECM with high content of fibrous proteins (mainly type II collagen) and water-binding, mostly sulfated glycosaminoglycans (sGAG) [35]. However, articular cartilage development is orchestrated by a cascade of molecular events regulated by various growth factors whose interactions allow the establishment of a well-designed architecture [34].

**Fig. 2:**
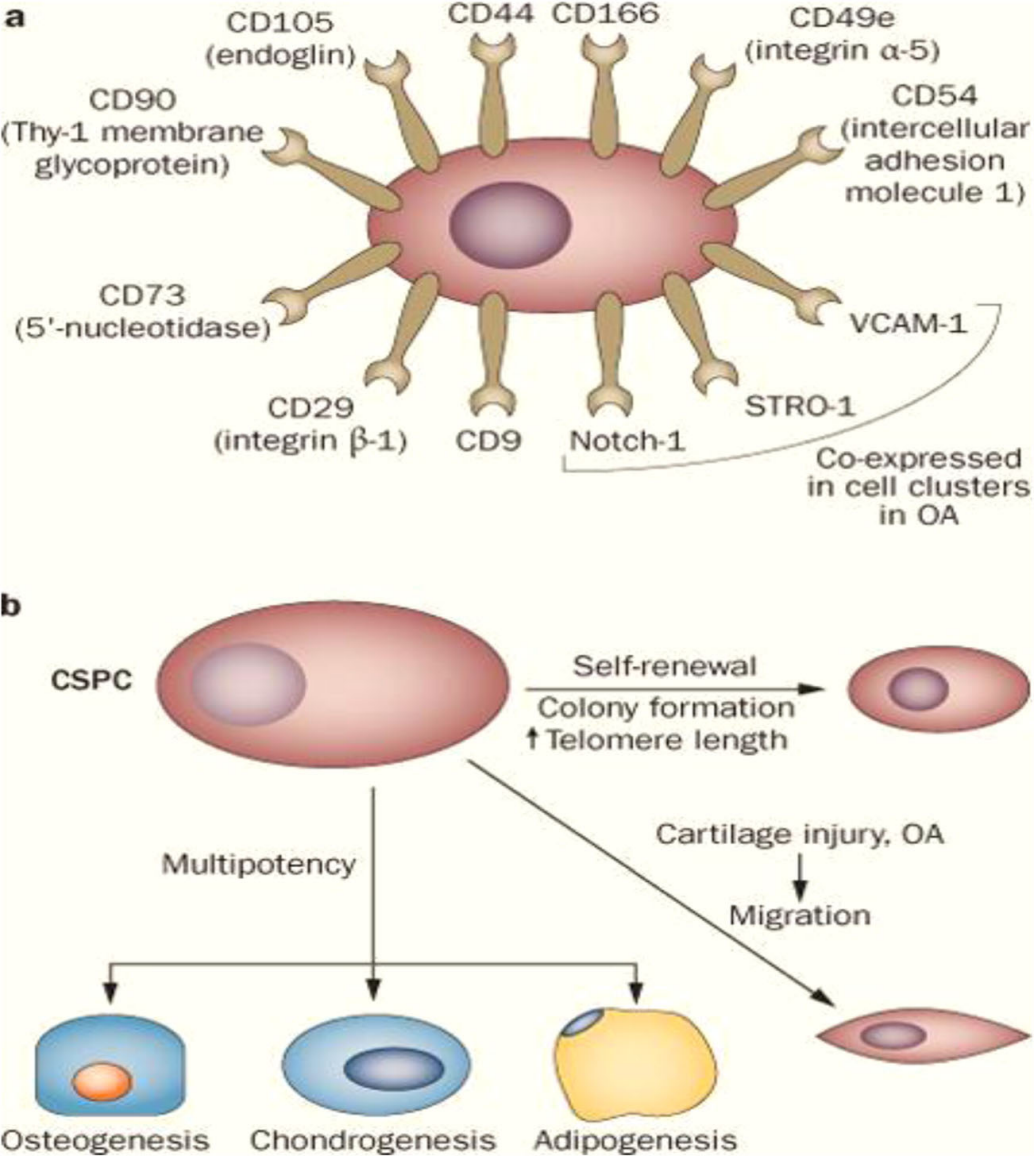
Characterization of CSPCs isolated from human, equine and bovine articular cartilage. **a** expression of stem-cell-related surface markers; **b** Properties of CSPCs and use for osteoarthritis (OA) management [41]

Integration of chondrocytes in the collagen network mainly composed of collagen type II, proteoglycans and glycoproteins results in the formation of cartilage tissue, since they act as binding proteins that will ensure the stabilization of the collagen framework. The resulting extracellular matrix (ECM) is characterized by a stability, which will ensure the rigidity and the resistance of the tissue when it is subjected to compressive and shearing forces. During the OC(D) pathogenesis, disruption of cell-matrix interactions plays a crucial role in the development of the disease, since it causes loss as well as superficial or deep fissures of collagen fibers [35]. When mature tissue injury occurs during the development of OC(D), the damaged cartilage has only a very low intrinsic self-repair and regeneration potential, as long as chondrocytes are considered to not have the capacity for migration, proliferation and repair. In fact, although they have low metabolic activity these cells are mainly involved in the production of key elements essential to maintaining the integrity of cartilage, namely: type II collagen and aggrecan, mainly known to play a fundamental role in the mechanical resistance of articular cartilage tissue [36–38]. Moreover, chondrocytes tend to lose their reproductive potential of the extracellular matrix (ECM) by dedifferentiation rather quickly. However, several studies have shown the presence of colony-forming cells in already dedifferentiated populations of human articular chondrocytes, with the ability to differentiate into chondrogenic, osteogenic and adipogenic cells, and co-expressing certain cell surface markers, such as CD105 (endoglin) and CD166 (activated leukocyte adhesion molecule). Being comparable to bone marrow MSCs in terms of their ability to differentiate, these cells were quickly referred as mesenchymal progenitor cells derived from cartilage [39–41]. It has therefore been proposed that the potentially reparative cartilage stem/progenitor cells could be involved in the processes of regeneration of damaged cartilage in the course of OC(D) [42]. Progenitor cells located in tissues are multipotent, highly clonogenic and chemotactic. During the appearance of a lesion, these cells will migrate locally to the damaged sites, where they will proliferate and differentiate according to the needs of the concerned tissue [43]. Like bone marrow stem cells, CSPCs have shown in-vitro a high capacity for self-regeneration by their ability to form colonies in culture [35], a length of telomere associated with significant telomerase activity [44], and typical characteristic of stem cells, a multilink power with differentiation capacity in osteogenic, chondrogenic and adipogenic cells [40]. These cells also present the same phenotypic characteristics of stem cells derived from bone marrow in humans and horses, in fact, various cell surface markers such as CD9. CD29 (integrin β-1), CD44, CD49e (integrin α-5), CD54 (intercellular adhesion molecule 1), CD73 (5′-nucleotidase), CD90 (Thy-1 membrane) glycoprotein), CD105, CD166, Notch 1 (homologous protein with neurogenic notch 1) and STRO-1 have been detected [41] (Fig. 2).

When OC(D) development induces the appearance of cartilage lesions, the stem / progenitor cells residing within the articular tissues (synovium for example), will begin to self-renew by an asymmetric division in order to initiate the formation of a pool of functional cells, which will regenerate and repair the damaged tissues [45]. Previous analysis of human OC articular cartilage has revealed the presence of CSPC cells expressing a number of markers specific to mesenchymal progenitor cells, including Stro-1, VCAM (CD166) and Notch-1; more interesting, tentative detection of the same cell type in healthy cartilage appeared to be negative. These observations therefore suggested that these progenitor cells may respond to recruitment stimuli from damaged cartilage, including dead tissue debris and chromosomal proteins [46]. Subsequently, several potential sources of MSC-like cells were explored, including mainly trabecular bone (epiphysis, metaphysis), bone marrow, periosteum, synovium, infrapatellar fat pad, synovial fluid, and skeletal muscle in the vital adult musculoskeletal system; and to a lesser extent, ligaments and tendons [47].

Furthermore, it has been reported that transcription factor 2 (Runx2) as well as the sex-determining region Y-box 9 (Sox9) play a crucial role in the processes of regulation and CSPCs cell mobilization. Therefore, Sox9 factor represents one of the fundamental elements regulating the synthesis of key components involved in the modeling of the cartilaginous matrix, in chondrocytes maturation and in the repression of chondral ossification process. On the other hand, Runx9 appears to be manly involved in regulating the development of osteoblasts and is therefore essential for bone development [48].

Apart from the fact that MSCs are stromal-derived cells, the latter have shown a strong ability to synthesize and regulate the different proteins that constitute ECM, as well as matrix methotroproteinases. Indeed, when these progenitor cells evolve in a chondrogenic environment, the latter initiate a differentiation process and expression levels of chondro-specific proteins, aggrecan and cartilage oligomeric matrix protein (COMP), collagen II and X, increase significantly, this phenomenon thus suggests that it would be more advantageous to use already differentiated stem cells to increase the efficiency of cellular therapy [49]. Recent advances in regenerative medicine have made MSC cells perfect candidates for the treatment of cartilage and bone diseases. Thus, lack of immunologically co-stimulatory cell-surface proteins expression and low expression of the major histocompatibility complex (MHC) II make these cells poorly immunogenic; and confer to them immunosuppressive and immunomodulatory properties, opening up great prospects in allogeneic MSC transplants [35].

Although exogenous supply of MSCs cells has shown great perspectives for the rescue of osteochondral and cartilage lesions, some studies have shown that endogenous repair systems involving endogenous progenitor and chondrocyte cells involved a much lower efficiency; thus, percentage of markers of cartilage derived mesenchymal stem cells (CD146 and CD166) significantly decreased in cartilage score with lower remodeling of cartilage tissue affected by OCD. In fact, these cells can, according to the degree of lesions severity, have a relatively low in-vivo mitotic potential under certain pathological conditions [50]. An attempt to repair cartilage lesions that have reached the underlying subchondral bone (osteochondral lesions) is however tentatively initiated, to form a primary mesenchymal blood clot, in the form of fibrocartilage [51]. However, this fibrocartilage is characterized by significantly lower biomechanical properties than hyaline cartilage and therefore has a greater fragility. Despite the fact that different experiments clearly demonstrated that the use of MSCs could greatly improve the repair process of damaged tissues, the therapeutic efficiency of these approaches did not correlate with the efficiency of engraftment, which appeared in the majority of cases to be relatively defective. These observations have suggested that the efficiency of regeneration depends on several factors, including the synthesis of various soluble biochemical mediators that can alter and modify the tissue microenvironment, and influence a wide range of biological functions such as inflammation. To the extent that existing data on tissue regeneration is controversial, and that the use of stem cells for cartilage regeneration is still in its infancy, more studies are needed to understand the role of MSCs in cartilage regeneration [52].

Another strategy in tissue engineering and cell differentiation is the use of pre-differentiated iPSCs in an MSCs-like population, to then, initiate their differentiation into chondrocytes [53]. IPSCs are a type of pluripotent and self-renewal progenitor cells that can be initiated from somatic cells by genetic reprogramming. Initially, four different factors involved in the molecular processes of differentiation were characterized in these cells, namely, the transcription factors 3 and 4 (oct3 / 4) binding the octamer, the Kruppel type factor 4 (Klf4), the homologue of the viral oncogene of avian myelocytomatosis (c-myc), and Sox-2. The derivation of iPSC from chondrocyte population can provide progenitor cells that highly express the gene encoding aggrecan [54]. It has also been shown that these cells express the majority of cartilaginous genes in a manner quite similar to that of chondrogenic cells. Thus, it seems that the use of iPSCs could be a new strategy for cartilage remodeling. Nevertheless, a certain number of axes have yet to be explored in order to optimize this type of biological technology, and to offer a better understanding of the biological mechanisms involved in the differentiation process, enabling the formation of productive and functional chondrogenic cell lines [55].

## Pathophysiological Mechanisms Underlying OCD Development

**Fig. 3:**
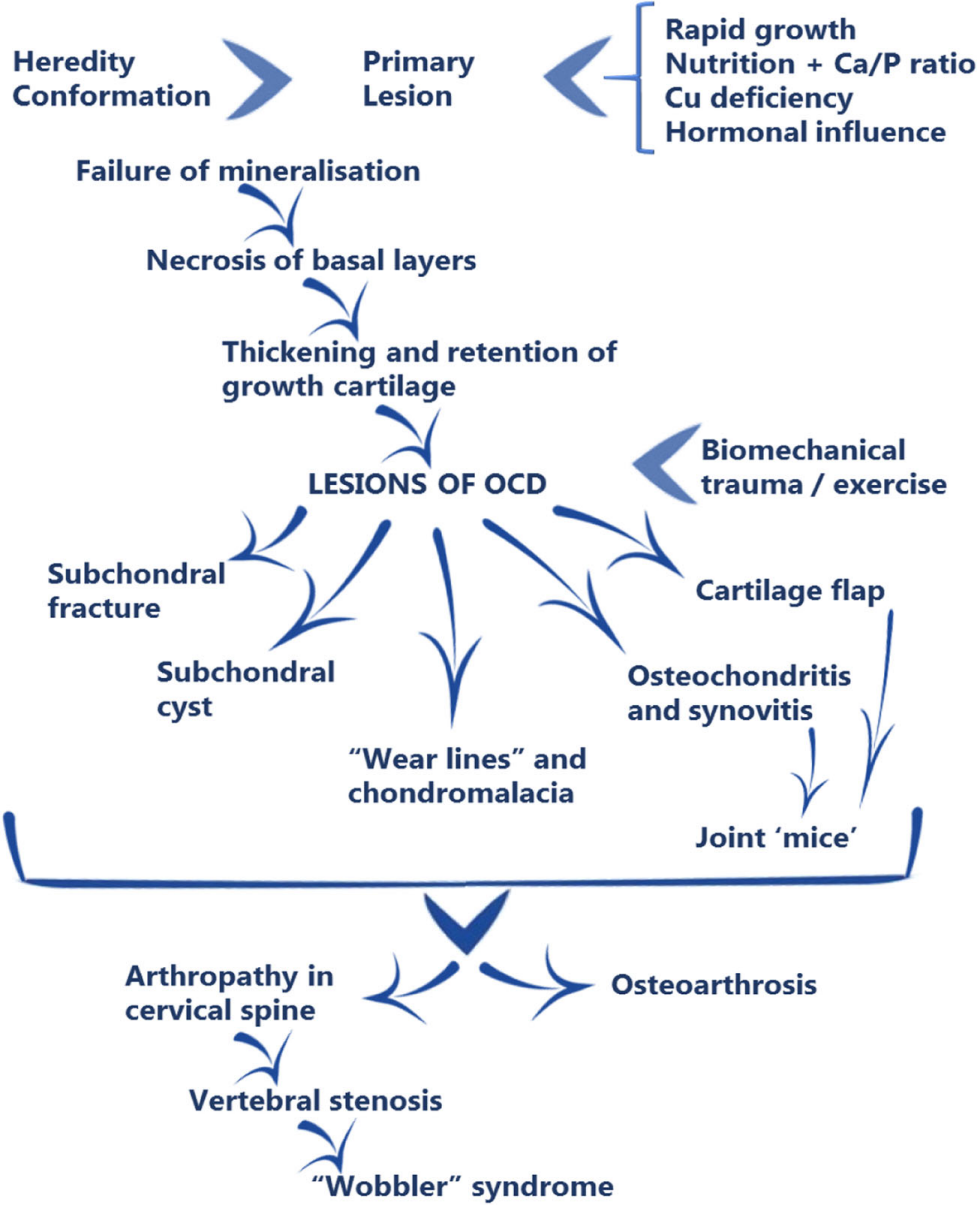
A schematic representation of the development of the osteochondrosis lesions complex in horses and the associated factors involved in pathogenesis [1]

During epiphyseal and metaphyseal growth plates development, OC causes a disturbance of endochondral ossification, and a subsequent failure of cartilage maturation resulting from the absence of penetrating capillaries into the hypertrophic growth plate zone. An interruption of endochondral ossification then occurs, leading to the retention of a thickened layer of cartilage. The cartilaginous complex of the growth plate is weakened due to avascular necrosis of the basal layers and the appearance of lesions manifesting as subchondral fractures, subchondral cysts and fractures of the cartilage flaps in case of OCD, binding link to detachments of joints cartilage fragments over time (Fig. 3) [56].

Although the involvement of several factors in the development of OCD is now widely accepted; in recent years, research has focused on the early pathogenesis of OC in horses to provide more details on its origin as well as the triggers. Thus, failure of the cartilaginous canals, biomechanical shear of the osteochondral junction, molecular changes in endochondral ossification, and genetic bases emerged as the most plausible causes [57].

Despite the exact origin of the disease seems to be of great complexity, a diagram of four main stages for OCD pathophysiological development can clearly be distinguished. During the first stage, OCD lesions begin to develop at the level of the subchondral bone where a large subchondral intraosseous osteopenia takes place. In a second time, these lesions will quickly be accompanied by an intraosseous oedema of the subchondral bone. The first trabecular microfactures of stage probably manifested as a bone bruise, may correlate with the oedematous morphological aspect of the bone marrow. The tissue thus injured will evolve into a sclerotic ring visible by radiology, delimiting the lesions of healthy bone tissue and bearing a center of lesions referring to osteonecrosis. On the other hand, the cartilage appears still intact at this stage. The evolution of the disease leads to subsequent softening and alteration of the mechanical properties of cartilage resulting into the release of an osteochondral fragment and the formation of a single loose body, or the detachment of several fragments, which are characteristic to OCD [58].

### Vascular Events in Early Osteochondrosis

The final phase of the endochondral ossification process is characterized by the invasion of the mineralized cartilaginous matrix layer by capillary buds and osteoblasts, leading to the gradual disappearance of chondrocytes, leaving room for osteoblasts that will form the mature bone tissue. It has been reported that during the course of osteochondrosis, blood capillaries are no longer able to penetrate the distal region of the hypertrophic zone, implying a consequent delay and a pathological modification of the cartilage maturation and the surrounding matrix; retention and thickening of the cartilage then occur, thereby weakening the entire cartilaginous articular / epiphyseal complex [59]. The appearance of the first latent lesions of OC(D) suggests that vascular failure occurs primarily at the time of integration of the vessels of the cartilaginous canal at the ossification front, this phenomenon being probably the result of microractures of the bone trabecular and trauma associated with vessels at this stage [60]. All OC lesions identified in horses aged 3 weeks to 5 months were consistently associated with necrotic cartilaginous vessels [2]. Postnatal failure of the cartilaginous canals along the ossification front is one of the most prominent causes in the development of OC(D) [61]. The defects in the anastomosis of the vessels of these channels lead to the appearance of focal areas of chondronecrosis. The development of this type of area accompanied by a delay in endochondral ossification and resulting in OC lesions in foals has previously been correlated with an existing disturbance of the joints blood supply. Indeed, the theory that vascular failure could be the cause of OC and OCD development has been explored experimentally by surgically sectioning vessels supplying the epiphyseal cartilaginous canals, from the lateral trochlear crest of the distal femur in Fjord pony foals aged 13 to 15 days. This vascular transection resulted in necrosis of the cartilaginous canals and chondrocytes, and pathological cartilage fracture in the area of ischemic chondronecrosis in all operated foals, leading to significant focal retardation in endochondral ossification. [62]. Other studies have shown that young foals presented signs of chondronecrosis due to disruption of the cartilaginous canal and a lack of blood supply [61]. The abnormal vulnerability of these vessels may be subject to instability of newly formed vascular anastomoses themselves, and to a weakness of the surrounding matrix, whose mechanical support appears to be markedly reduced. Moreover, it is admitted that ossification front is a metabolically active region subjected to matrix proteolysis; it is also a transition region between tissues (growth cartilage and bone) with different mechanical properties. Vessels crossing this area are potentially subjected to greater biomechanical constraints. Direct mechanical shear of the cartilaginous canals at these sites may explain the distribution of OCD lesions [17].

### Cartilage Matrix Changes

During growth, the cartilage undergoes a series of complex cellular events ensuring the transition from the cartilaginous matrix to the skeletal maturity, this process notably involves the proliferation of chondrocytes, maturation, hypertrophy and mineralization of the ECM prior to the invasion of the cartilage by blood vessels [63]. Denaturation of extracellular matrix (ECM) collagen plays a pivotal role in the initiation of OC; thus, an exacerbation of the type II collagen solubility has been observed in the cartilage of foals with OC, pointing out that abnormalities of collagen metabolism of articular cartilage, are fairly involved in the onset of OC as previously reported in many species including humans, pigs, and horses [17, 64]. Moreover, it has been demonstrated in an experimental model of OC as well as in the course of OCD of natural origin, that significant reduction in total collagen II content accompanied with cross-link rupture were predominant in early lesions [65, 66]. Probable imbalance between the different enzymes involved in proteolysis of the cartilaginous matrix at the ossification front is also one of the factors that can contribute to the development of OCD. An increase in intracellular lysosomal enzyme levels, cathepsin B, being able to degrade both type II uncoiled collagen and proteoglycan, has already been identified in equine OCD lesions [67]. In addition, analysis of cartilaginous explants bearing OCD lesions as well as serum and synovial biomarker measurements of animals suffering from OCD, revealed a significant increase in collagen turnover [68]. One of the main structural components of cartilage is type II collagen, which contributes to the maintenance of the joint’s biomechanical functions such as resistance to increasing pressure exerted by cartilage proteoglycans or shear stresses generated during joint movement, thanks to its spatial 3D conformation organized in network of collagen fibers [69, 70]. Significant reduction in the number and thickness of collagen fibrils has been demonstrated in areas of ischemic chondronecrosis in pigs, this has been attributed to the inability of chondrocytes to perform their role in homeostasis of the cartilaginous matrix [71]. Spectacular structural changes of collagen have been reported by Lecocq et al. [68]. The usually dense collagen network has been found to be much thinner in the ossification front in equine tissues, concomitantly with pathological cellular changes represented by increased cell volume, cell death, proteolysis of collagen and thus the reduction of its content. This would contribute to the structural weakening of the articular cartilage and could predispose to micro or macro-traumatic lesions, lesions of the blood vessels and the subsequent development of OC(D).

### Molecular Events

When alterations occur in the articular cartilage, meniscus, ligament or synovial membrane, some molecules may be released into the synovial fluid, whereas the biomarkers of bone tissue will generally be released into the blood, if the underlying bone of a joint is involved [12]. In order to elucidate the molecular bases of OCD development, several investigations have focused on the study of the different biochemical changes that may appear during joint diseases, as well as the distribution pattern of main matrix molecules (Type II collagen, cartilage oligomeric matrix protein, large-aggregate proteoglycans, fibronectin, cartilage matrix protein and biglycan) following the development of early lesions resulting in necrosis of the epiphyseal cartilage [4]. In addition, significant changes in cartilage turnover markers involved in the metabolism of collagen and proteoglycan have been demonstrated during the analysis of synovial fluid or serum of foal with OCD [66, 72].

#### Matrix Metalloproteinases (MMPs)

The matrix metalloproteinases represent a large family of more than 22 different zinc-dependent endoproteases, whose main function resides in the physiological turnover of the extracellular matrix (ECM); these enzymes have also the ability to degrade all the components of the ECM cartilage [73]. Depending on the nature of their natural substrates, four main groups of MMPs can be distinguished; the first comprises collagenases (MMP-1, 8 and 13), which are responsible for the cleavage of the interstitial collagen triple helices; MMP-2 and -9 gelatinases, that degrade unwound collagen and gelatin; the endoproteases MMP-3, −10 and − 11, which are stromelyses responsible for the degradation of collagen type II, IX and XI, proteoglycans, which can act as activators of pro-MMP-1, −8, −9 and − 13. Finally, membrane-type MMPs (MTMMPs) activators of different pro-MMPs such as MMP-14, −15, −16 and − 17 [74, 75]. Numerous studies have already clearly demonstrated the major involvement of MMPs in the pathological degradation process of cartilage during various forms of arthritis [76–78]. Significant increase in the production of these enzymes has been observed in rheumatoid arthritis joints [79, 80] and osteoarthritis cartilage [81]. A consequent elevation of MMP-9 levels measured in the synovial joint fluid with severe cartilaginous alterations in different joint pathologies (OCD, DJD and ESF) was already observed, and occurrence of the active form of the MMP-9 monomer was associated with the presence of a degenerative process [82]. Additionally, hyperactivity of MMP-2 was detected by the mean of gelatin Zymography in synovial fluid of OCD-bearing horses in comparison with synovial fluids from horses with no diagnosed joint disease [83]. The mechanism underlying the MMP-2 over-activity remains unclear but could involve defective regulation by TIMP-2, which is the main inhibitor of MMP-2, or a defective expression of TIMP-2 [82, 84]. Moreover, an increase in serine protease levels has been demonstrated in OCD articular cartilage [67]. MMP-3 also plays a fundamental role in the destruction of the cartilage matrix since it is responsible for the degradation of several cartilage components and is essential for the activation of other MMPs. Differential analysis of damaged and healthy joints cartilage showed that MMP-3 activity as well as its concentration in the synovial fluid significantly increases with the increase of the cartilaginous lesions’ severity degree, thus suggesting the implication of this enzyme in the cartilage degeneration during equine OCD [85].

#### Cartilage Oligomeric Matrix Protein

Another presumed marker for OCD is COMP. This non-collagen homopentameric protein with 5 globular domains, attached to a central assembly domain by flexible strands belongs to the thrombospondin family. Found almost exclusively in the extracellular matrix of load resisting tissues, it is considered to be one of the most important proteins for the formation and stability of the extracellular matrix, thanks to its ability to complex with fibronectin and type I, II and IX fibrillar collagens and its participation in the organization of collagen fibrils in networks [86]. Several investigations have already pointed out the implication of this protein in various bone conditions including age, osteoarthritis, OCD and joint sepsis. COMP concentration in synovial fluid has been shown to decrease with osteoarthritis in horses [87–89]. Significant concentrations of COMP have also been reported in the synovial fluid of Thoroughbreds with osteochondral fractures compared to Standardbred trotters with osteochondral fractures and Thoroughbred and Standardbred trotters with OA. This increase in COMP in synovial fluid appears to be correlated with post-traumatic duration, suggesting that COMP may also be an appropriate marker for longitudinal studies to evaluate its role in joint healing [90].

#### Peptide of the α-Helical Region of Type II Collagen (Coll2–1)

Recently, some markers of collagen degradation such as Coll2–1 have been introduced into human and equine medicine for evaluation of joint disease [91]. Coll2–1 is a peptide ( [92]HRGYPGLDG [93]) located in the triple helix of the type II collagen molecule, and is specific to joint diseases since it is a component of collagen only. In various pathologies involving joints such as OC(D) or OA, collagen type II, which represents the main structural protein of cartilage constituting about 50% of the extracellular cartilage matrix, is generally degraded by enzymatic and mechanical actions, leading to the release of fragments in the synovial fluid such as Coll2–1 [94, 95]. Synovial levels of Coll2–1 in OCD and normal tarsocrural joints in horses have been previously analyzed. Horses with OCD had significantly higher Coll2–1 synovial levels compared to healthy horses, indicating the progress of a cartilage degradation process in these injured joints; it has therefore been postulated that Coll2–1 could be an earlier and more sensitive marker than C2C, a marker conventionally used for the detection of cartilage degenerative lesions in horses [96].

#### Osteocalcin

Osteocalcin, or bone Gla-protein (BGP), is a small protein synthesized by osteoblasts and odontoblasts, containing carboxyglutamic moieties and constituting the major part of non-collagenous bone matrix. Many studies demonstrated that serum osteocalcin provides a useful marker of bone metabolism in several bone diseases [97]. During the mineralization process, calcium phosphate is first deposited in matrix vesicles derived from cells dispersed throughout the hypertrophic zone, before being successively converted to hydroxyapatite. Furthermore, the rate of crystal proliferation is favoured by the abundance of calcium, phosphate, collagen, and delayed by the proteoglycans and non-collagenic proteins fixing calcium such as osteocalcin. Overproduction and hyperactivity of mineralization inhibitors, mainly represented by osteocalcin leads to defective calcification and cartilage retention resulting in OC(D) changes and damages [50]. Moreover, serum osteocalcin concentrations were found to be significantly correlated with the severity of osteochondrosis in foals during the first year after birth [98].

#### Chondroitin Sulphate 846 (CS-846)

Chondroitin sulphate is a component of cartilage proteoglycans. The chondroitin sulfate 846 (CS846) epitope is located on the chondroitin sulfate side chains near the G3 domain, and as such represents newly synthesized aggrecan molecules; it is released from the extracellular matrix into the synovial fluid once it is cleaved from the aggrecan protein. These large foetal forms of aggrecan are naturally present in young animals, but then decrease in cartilage with age [12]. Chondroitin sulfate 846 (CS846) is an inseparable marker of the degree of joint injury in several cases of DOD. CS846 with glycosaminoglycan (GAG) are direct biomarkers of proteoglycan degradation in the cartilage matrix. Particularly high CS-846 levels found in serum and synovial fluid linearly correlated with severity grade of osteochondral lesions, indicate that this epitope is closely associated with OC(D) and suggest involvement of increased synthesis of cartilaginous aggrecan and procollagen type II during the pathophysiological development of the condition [99].

## Impact of Oxidative Stress in the Course of OCD Development

Reactive oxygen species (ROS) can be responsible for the degradation of several key components of articular cartilage, notably collagen, proteoglycans and hyaluronan [100]. When a joint trauma occurs, cellular disturbances and subsequent phagocytic activation stimulate the production of ROS, which exacerbates traumatic tissue damage [101]. As a result, ROS appear to be widely involved in the pathogenesis of joint diseases in humans, but also in athletic horses [102, 103].

Local ischemia (restriction in blood supply to tissues), which affects epiphyseal cartilage in the articulo-epiphyseal cartilage complex, initiates the formation of highly vulnerable necrotic epiphyseal cartilaginous zones, responsible for the underlying delay in endochondral ossification, where necrotic cartilage will tend to infiltrate subchondral bone [3]. The oxidative phorphorylation provided by the mitochondria is seen to be significantly reduced during ischemia, this leads to a decrease in the level of produced cellular ATP. To remedy this, cells will metabolize more excessively AMP-mediated molecules via the purine pathways of hypoxanthine and xanthine. Upon reintroduction of molecular oxygen into the tissues by reperfusion or in the event of microfractures, hypoxanthine and xanthine oxidase will reduce molecular oxygen to superoxide radical [102]. In the course of superficial cartilage samples study obtained from OCD horses, an increase in the cell population having undergone apoptosis process and being immuno-positive for nitrotyrosine, an indicator of increased production in NO have been detected. Hypothetically, injured cartilage is characterized by the presence of microfractures responsible for a wider exposure of the tissue to the synovial fluid. This phenomenon causes the diffusion of oxygen and nutrients through the cartilage. Chondrocytes, which until then were essentially anaerobic cells, will tend to redirect their metabolism to a catabolic pathway. This will trigger a cartilage remodelling that leads to the production of cytokines like IL-1 by resident and inflammatory infiltrating cells; the release of IL-1 will then inevitably initiate the biosynthesis of nitric oxide (NO) by the articular chondrocytes. Consequently, NO will integrate the catabolism of the cartilage and stimulate the production of other free radicals, and initiate radical lesions of the cartilaginous matrix, this in addition to a possible intracellular action on the signalling pathways of the expression of certain genes, will finally induce the death of the neighbouring chondrocytes through apoptosis [104]. Oxidation of proteins by free radicals results in the formation of carbonyl groups at the amino acid residues, and therefore in their denaturation. Assessment of total carbonyl derivatives content in synovial fluid samples collected from OCD-related horses joints, revealed increased protein oxidation compared to clinically healthy control groups; thus, high levels of carbonyl protein in the cartilage of diseased horses necessarily implies an abnormally high presence of ROS in the joint, and thus suggests the involvement of oxidative stress in joint damage [105]. In addition, the presence of high concentrations of ROS was correlated with the sharp decrease in the viscosity of hyaluronan, leading to the loss of its elasticity and the onset of increased friction of the joints. Moreover, the decomposition of hyaluronan into lighter molecular chains resulting from the damage caused by free radicals makes it lose its physiological functions; the latter being no longer able to inhibit phagocytosis or production of associated ROS, the oxidative stress will tend to exacerbate within the joint [106]. Many other components of ECM may be the target of ROS; and collagen is one of the most severely affected component. Collagen is naturally designed to form a characteristic gel under physiological conditions. The presence of excess superoxide in tissues promotes the breakdown of collagen by splitting the non-helical regions and initiating the oxidation of critical amino acids, and more particularly the methionine, tyrosine and histidine residues, which are necessary for the aggregation process [107].

## Mitochondrial Dysfunction and Endoplasmic Reticulum Stress

Recently, abnormalities in mitochondria and endoplasmic reticulum of deep cartilaginous tissues have been detected. It has been postulated that disturbance of the Wnt signalling pathway, a key regulator of mitochondrial function, typically characteristic of OC equine cartilage may be partly responsible for mitochondrial dysfunction [108].

The Wnt signalling pathway, which comprises numerous proteins, receptors and inhibitors, takes part in various physiological processes and in particular, in the regulation of proliferation, maturation, differentiation and endochondral ossification of chondrocytes. Thus, analysis of the genetic profile of leukocytes sampled from OC-horses revealed an alteration of the canonical and non-canonical Wnt signalling pathways [29]. Overexpression of the b-catenin and Lrp6 genes promoting the canonical and non-canonical pathways (Wnt-5b) seems to highlight their involvement in the pathogenesis of early OC lesions [109]. Furthermore, it has been previously demonstrated that Wnt signalling pathway strongly activates mitochondrial biogenesis, resulting in further modulation of free radicals production and subsequent onset of oxidative stress. This exacerbation of highly reactive ROS generation via mitochondrial dysfunction may be responsible for some of the major biological consequences of aberrantly activated Wnt signalling, such as stem cell depletion or early osteochondral lesions [92]. A number of important pathologies as well as altered biological processes have been associated with mitochondrial swelling, mainly responsible for overproduction of ATP and apoptosis [110]. Aberrant activation of the respiratory chain resulting in abnormal production of ATP has been implicated in the phenomenon of increased volume of the cartilage matrix [111]. Chondrocyte hypervolemia observed during OCD might represents an adaptive mechanism against the energetic stress resulting from altered cellular machinery, where the down-regulation of proteins involved in the mitochondrial function is prominent. Analysis of osteochondral cartilage samples also showed a down-regulation of several proteins involved in mitochondrial function, such as LGALS3, AK4, HEBP2, SSBP1 and CYB5A involved in energy production, with ATP5C1 and ETFA playing a role in electron transport; and PRDX5 on oxidative stress. Homeostasis and chondrocyte differentiation are regulated by, among other things, energetic production, thus the involvement of mitochondrial dysfunction in the pathophysiological process leading to joint abnormalities seems to be a plausible pathway, with a possible impact on ECM catabolism and calcification, protein biosynthesis, release of pro-inflammatory cytokines or apoptosis of chondrocytes [112]. Proteomic study of proteins involved in energy production, organization of the mitochondrial membrane and detoxification of free radicals has identified significant differences in their expression between healthy cartilage and human arthrotic cartilage [113].

Accumulation of misfolded proteins and disruption of endoplasmic reticulum functions occur in many diseases including dyschondroplasia [114]. Constitutive abnormalities in the proteosome-dependent degradation process of damaged or misfolded proteins in the ER were detected during OCD in horses. For this purpose, down-regulation of PSMA, one of the major components of the proteasome complex and PSMD14 has been highlighted. In addition to the repressing effect of ER on the synthesis of damaged proteins, it can also affect various other cellular processes such as calcium signalling pathways, biosynthesis and lipid uptake [115]. Down-regulation of several associated proteins in calcium homeostasis (S100A1, S100A2…) observed during OCD suggests involvement of ER stress in alteration of synthesis and repair of cartilage matrix [116]. One of the main constituents of articular cartilage is aggrecan, a proteoglycan consisting of a large protein core richly substituted with sulfated glycosaminoglycan (GAG) chains. The core of this protein is synthesized in the rER and then trans-localized into the Golgi for maturation before integrating the ECM structure. Misfolded aggrecan molecules accumulated in the rRE have thus been localized in osteochondrosis cells, these alterations have subsequently resulted in ER stress and pathological disruption of the cellular protein synthesis. The ultimate consequence of this damage lies in the development of an abnormally structured cartilaginous ECM [93]. It was also reported that accumulation of fibril bundles composed of abnormally wide type II collagen heterofibrils, and abnormal matrix accumulation within dilated rER, together with circular forms of abnormal collagen filaments in ECM, may result from abnormalities in the procollagen assembly and/or defective fibril formation (cross-linking), and leading to permanent dedifferentiated chondrocytes [117].

## Management of OCD

The joint is an organ regularly subject to various traumatic lesions, leading to a dramatic and definitive degradation of the cartilage, which resides therein. Rapid management of synovitis and capsulitis is an essential step in the medical approach to reduce or prevent further degradation of the cartilaginous matrix. The treatment of articular traumatic entities is inscribed with the aim of first bringing the joint back to its natural state in a quick and effective manner, and secondly preventing the occurrence of more severe complications that can lead to the destruction of articular tissues. In the case of OCD, medical treatment will focus on removing fragments of osteochondral chips, reducing predominant intra-articular fractures and accurately diagnosing ligamentous and meniscal lesions [118].

The most widely adopted treatment strategy for the treatment of OCD in horses is the surgical debridement of osteochondral lesions. However, when minimal lesions affect foals at early age and do not induce any clinical signs of concern, prolonged rest and radiography are sufficient to remedy the condition. Arthroscopic surgery is therefore still necessary for the most serious cases, where animal lameness and predominant swelling of the affected area can be observed. During the procedure, affected joint is minutely explored in order to detect all possible lesions. All parts of the damaged joints will then be removed (loose tissues and bodies). Debridement is then performed until only healthy tissue remains. Special attention should be given during the debridement of the young foals to avoid damaging of the subchondral bone, which is still soft at this stage [119].

Another approach with dual objectives, curative and preventive, consists of nutritional management. The diet of foals must include limited rations of easily digestible carbohydrates to limit the risk of hormonal imbalances and also regulate the rate of growth of young animals. It has also been suggested that copper supplementation (an oral dose of 0.5 mg copper sulphate / kg body weight / day) should be incorporated into mature mares raised in areas where the natural availability of copper is too low. It would be quite convenient to ensure a pasture access for foals throughout the growing stage [120]. The other therapeutic options that can be used to treat OC early lesions, mainly consist in reducing the intensity and frequency of exercises of affected horses and foals. On the other hand, the use of medication is sometimes unavoidable, especially during the onset of synovitis, where intra-articular injections of hyaluronic acid are quite common. When some foals have larger cartilaginous flaps that are relatively unrelated to the subchondral bone, arthroscopically placed polydioxanone needles (PDS) can be used to attach the flaps to the underlying subchondral bone [121]. Moreover, recent scientific advances in this field have led to the development of new treatments based on mesenchymal stem cells, platelet-rich plasma and various other biological entities that can be adapted according to the type of lesion (OCD) [16]. Some investigations have already focused on the evaluation of the regenerative potential of new biomaterials on different equine cellular models; however, very few of them have been devoted to studying the impact of this type of strategy on the treatment and repair of lesions and other osteochondral affections resulting from a pre-existing injury or pathology such as OCD. For this purpose, an attempt to repair a dissecting osteochondral fibrocartilaginous lesion was performed using a bio-dispositive consisting of a gelatin β-tricalcium phosphate sponge, impregnated with platelet-rich plasma, bone morphogenetic protein-2 and mesenchymal stem cells combination [122]. Currently, literature reports on experimental mesenchymal stem cells transplantation shows that their activation in defected cartilage using various scaffolds in the presence of combined growth factors including TGF-β, BMP-2, BMP-4 and PDGF has led to greater improvement in lesion repair and tissue regeneration, compared to scaffolds without any growth factors supplementation. However, complete and effective long-term cartilage regeneration has not yet been achieved using these methods, suggesting that there is still a lack of understanding of the growth factors role and their optimal combination in the differentiation process of stem cells [123].

## Conclusion

Osteochondritis dissecans (OCD) is a relatively common developmental disease that affects the cartilage and bone in the joints of young horses of different breeds, with increasing incidence. The primary osteochondrosis lesions are directly associated with focal failure of the endochondral ossification. Since osteochondrosis is known as a multifactorial condition, several theories on the pathophysiological mechanisms underlying the development of the disease have been proposed, the majorities of which, have already been proved by relevant scientific works, such as traumatic lesions, ischemia and failure of cartilaginous canals and blood vessels, dyschondroplasia and alteration of the cartilaginous matrix, accompanied with defects in expression of several genes as well as various cellular biological processes, have been clearly evidenced to take part in the pathogenesis of OC(D). Although the molecular aspect is more and more explored for a better understanding of the pathology, some tracks such as implication of the mitochondrial machinery, oxidative stress or other associated pathologies including insulin resistance, obesity and metabolic disorders have not yet been considered for the complete elucidation of the molecular basis of OC(D) pathogenesis and the subsequent development of more suitable treatments.
